# Probing the Coupled Equilibria between Metal Nanoparticles, Antibiotics and Components of the Extracellular Matrix in Biofilms with SERS

**DOI:** 10.1021/acs.biomac.4c01707

**Published:** 2025-04-07

**Authors:** Wafaa Aljuhani, Matthew P. Wylie, Rudra N. Purusottam, Colin P. McCoy, Steven E. J. Bell

**Affiliations:** † School of Chemistry and Chemical Engineering, Queen’s University Belfast, Belfast BT9 5AG, U.K.; ‡ School of Pharmacy, Queen’s University Belfast, Belfast BT9 7BL, U.K.

## Abstract

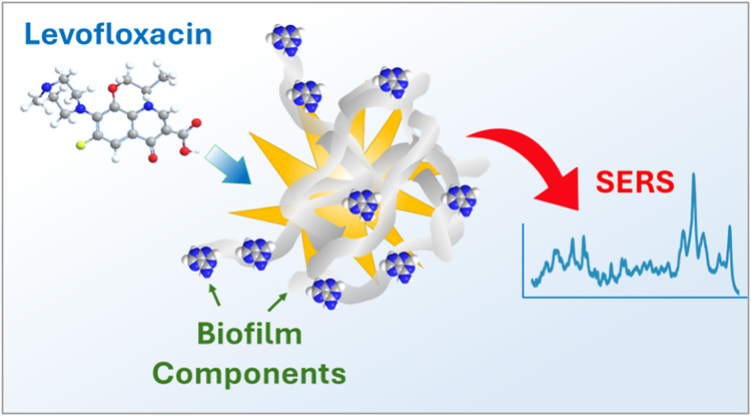

Understanding the interplay between nanoparticles, biomaterials and drug molecules in biological environments is important but studying these interactions in complex systems such as biofilms is challenging. Here, surface-enhanced Raman spectroscopy (SERS) with gold nanostars (NS) was used to monitor how biofilm components influence the binding and SERS signals of two antibiotics, levofloxacin (Levo) and ampicillin (Amp). The SERS signals of both antibiotics were reduced by approximately 70% (Levo) and 90% (Amp) in biofilm environments. Investigations of mixtures of model biofilm components: adenine (nucleic acids), alginate (polysaccharides) and albumin (proteins), revealed that their interactions with NS are governed by coupled equilibria. This gave surprising results, for example, alginate reduced the interference from adenine and albumin, so adding alginate increased the intensity of the antibiotic signals 4x. These findings highlight the importance of matrix component interactions in modulating detection sensitivity and show that these effects are critical in allowing clinically relevant detection levels to be achieved.

## 1 Introduction

Understanding the intricate interplay between complex biomaterials and nanoparticles in biological environments is important because such interactions have significant implications for applications that range from the development of drug delivery systems to the design of antimicrobial surfaces.^1^ Similarly, understanding the interaction between drug molecules and nanoparticles within biological environments is critical for applications such as therapeutic drug monitoring.^2^ However, it is challenging to study such interactions in real biological environments since these are typically rich in molecules capable of interacting with nanoparticles such as proteins, ions, lipids and nucleic acids, making it difficult to isolate and understand specific interactions.^3^

One area where these considerations are particularly obvious and challenging is biofilms. Biofilms are complex systems because they consist of microbial populations encased in a self-produced extracellular matrix (EPS) primarily composed of polysaccharides, proteins and DNA.^4^ Conventional methods for studying biofilms include microtiter plate assays for quantifying biofilm formation, and confocal laser scanning microscopy (CLSM), mass spectrometry imaging (MSI) and scanning electron microscopy (SEM) for imaging of the biofilm.^5^ However, there is a real need for a simple approach to analyzing biofilms which combines molecular specificity with high sensitivity and the possibility of carrying out imaging studies. Surface-enhanced Raman spectroscopy (SERS), which utilizes the interaction of plasmonic nanoparticles with light to enhance the Raman signals of molecules near the nanoparticle surface,^6^ could be such an approach.

SERS is already emerging as a powerful tool for rapidly studying biological environments in real time. At the simplest level it has been used for the characterization of pure samples of biomolecules such as lipids, proteins and nucleic acids, providing insights into their chemical composition and structure.^6,7^ However, it can also be used to probe intact tissue, bacteria, cell cultures etc. as shown in numerous previous studies.^8,9^ The affinity which many biological molecules and biopolymers show for metal nanoparticles is an advantage in work where the objective of the SERS studies is to detect these adsorbing species since it brings them onto the enhancing surface. However, it can be a significant problem if the intention is to detect other materials or compounds since adsorption of the matrix components can reduce the binding of the target to the surface and therefore the signals they generate.^8^ Of course the simplest approach may be to purify the biological sample, for example by removing interfering proteins but this is not possible for *in situ* or *in vivo* studies. This means that it has been necessary to develop other methods for reducing the matrix interference. Notably, modification of the surface with PEG is often used to reduce protein adsorption and has been used in SERS measurements of fentanyl in blood plasma for example.^10^

In the case of biofilms, the nature of the extracellular matrix makes SERS methods particularly challenging since it is chemically complex, contains multiple constituents known to interact strongly with metal nanoparticles and has a nonuniform gel-like structure.^4^ The most successful previous SERS studies have used solid substrates to monitor biofilm growth and production of strongly SERS active quorum signaling molecules at the surface of the supporting substrate.^11^ In the current study the objective is to record data from within the biofilm, which requires the use of nanoparticles as the plasmonic enhancing media, since these can be dispersed within the extracellular matrix, rather than sitting at the surface. This means that the matrix effects need to be addressed directly.

In this work we use the approach previously developed for removing the effects of the inhomogeneity and gel-like structure of the biofilms by working with *ex-situ* biofilms (es-biofilm), where biofilms are diluted and extracted from culture plates to obtain a homogeneous controllable liquid which has the same overall composition as the intact parent biofilm samples. This was combined with the use of nanostars (NS) which were chosen since they are plasmonically active without being aggregated in the way that is necessary for conventional spherical or quasi-spherical particles and are stable in *es*-biofilm.^12^ This approach has allowed us to explore the extent to which SERS can actually be carried out in these very complex matrices and if the use of the NS enhancing particles will allow measurements with useful levels of sensitivity and molecular specificity to be made. This approach can open up a range of possibilities, the most obvious would be the detection of drug compounds within the biofilms. This is an important problem because 80% of all human bacterial infections are linked to biofilm formation and monitoring how antibiotics move through the extracellular matrix is critical in understanding how the biofilm increases resistance to antibiotics.^13^ However, this is a small part of a much larger question, which is how the components within the biofilm interact with each other and with both the metal nanoparticles and drug molecules? Some parts of this complex picture are already well-known, for example, SERS has been employed to investigate interactions between biomolecules, such as protein–DNA and protein–lipid interactions.^14,15^ In addition, the physical structure of the EPS depends on interactions between the e-DNA and biopolymers within the matrix.^16^ However, the possibility of multiple simultaneous interactions between several of the components is rarely considered.

In this study, we have found that NS can be used to detect important antibiotics, levofloxacin and ampicillin, at clinically relevant concentrations in *ex-situ* biofilm, although the sensitivity of the measurements was found to be significantly compromised in comparison to simple aqueous solutions. Further investigations of the impact of individual biofilm components, including proteins, polysaccharides and adenine, allowed the effect of each of these components on detection of the drug molecules to be determined. Surprisingly, it was found that the effects in multicomponent model systems were not simply additive and that these complex systems could be best described in terms of a model involving multiple coupled equilibria between each of the components present. In some cases, interactions between species could be inferred from the SERS data, even if it was not directly observed but ^1^H NMR experiments for example provided direct confirmation of interactions between alginate (the model polysaccharide) and adenine (nucleic acid).

These results are important because they directly address the challenges of monitoring drug compounds in biofilms with metal nanoparticle sensors/probes. However, they also have a broader significance because they demonstrate that the interactions of nanoparticles with biological systems (which typically consist of multiple interacting components) can be significantly perturbed by the interactions between the constituents. This means that models of these complex systems which are based entirely on simple equilibria of individual constituents and metal nanoparticles are inherently incomplete and may be misleading.

## 2 Methods

### 2.1 Sample Preparation

Nanostar colloids (NS) were prepared using a previously reported method.^17^*Ex-situ* biofilms were also prepared using a previously established protocol.^18^ In brief, an overnight culture of *Staphylococcus aureus* (*S. aureus*, ATCC 29213) was diluted in Tryptic Soy Broth (TSB) to achieve an inoculum concentration of 10^6^ CFU/mL. Then, 150 μL of this inoculum was added to a sterilized 96-well microplate (Thermo Scientific) and incubated at 37 °C and 100 rpm for 24 h. After this period, the broth was replaced with 150 μL of fresh sterile medium, followed by another 24-h incubation under the same conditions. At 48 h, the medium was removed, and each well was rinsed three times with sterile water to eliminate any remaining medium and unattached cells. Then, 5 μL of sterile water was added to each well and the plate was sonicated for 15 min to release the biofilm from the well walls into the water. The *ex-situ* biofilm solutions from each well were then collected into a sterile container for SERS analysis.

### 2.2 Characterization

The size distribution of the NS colloids was obtained using Nanoparticle Tracking Analysis (NTA) with a Malvern NanoSight NS300. UV/vis extinction spectra were recorded using an Agilent 8453 single beam diode array spectrometer.

Transmission electron microscopy (TEM) imaging of the NS was performed on a Joel JEM-1400 Plus Transmission Electron Microscope and a TALOS F200X G2: Scanning/Transmission Electron Microscope (S/TEM). The sample was prepared for TEM by depositing a drop of the colloidal solution onto carbon-coated grids (S160, 200 mesh Cu (25)) and allowing them to dry at room temperature.

### 2.3 SERS Analysis

Measurements were conducted using an Avalon RamanStation R2 benchtop Raman spectrometer equipped with a 785 nm diode laser operating at 160 mW power (10 × 10 s accumulation time). The spectra are presented with minimal preprocessing, limited to intensity scaling for display purposes and averaging to ensure more reliable and consistent results.

In all experiments, 25 μL of the test sample (biofilm, adenine, alginate, adenine-alginate mixture, albumin or albumin-alginate mixture) was added to 175 μL of the colloid. After allowing the mixture to react for 3 h, 25 μL of drug solution was added. This resulted in a final drug concentration of 10^–3^ mol dm^–3^ and, where applicable, a final adenine concentration of 10^–5^ mol dm^–3^, a final alginate concentration of 0.1% (w/v) and a final albumin concentration of 0.1% (w/v).

For quantitative comparison of the effects of added constituents the results measurements were repeated four times using the same batch of colloid and concentration and the results averaged.

For the experiments evaluating the binding of alginate to the nanoparticles to displace the citrate. Twenty-five μL of 10^–3^ mol dm^–3^ cit rate was added to 175 μL of colloid and allowed to react for 5 min, then 25 μL of 2% alginate (w/v) was added and the spectra were obtained. Alginate-colloid and citrate-colloid samples containing the same concentrations of alginate and citrate as used in the mixture were also prepared.

### 2.4 NMR Analysis

All NMR experiments in this study were conducted using an Avance Ascend Bruker 600 MHz spectrometer. The instrument was operated at 298 K and was equipped with a TXI probe head. The spectrometer used the noesygppr1d pulse sequence (Bruker, size of fid 65536, 12406.95 Hz spectral width, 128 scans and d1 = 4 s) for recording ^1^H one-dimensional experiment with presaturation delay during relaxation and mixing time. Using this pulse sequence allows for the detection of all nonexchangeable protons in a deuterated buffer solution, making it highly effective for detailed molecular analysis. The total experimental time for this pulse sequence was 14 min and 42 s and Bruker Topspin version 3.6.5 software was used for data acquisition and analysis. The concentrations of the samples of pure adenine and alginate were 0.1% and 0.5% (w/v), respectively. In the mixed samples, the higher solubility allowed a higher concentration of adenine and alginate to be prepared to maximize the probability of observing interactions between the two components.

## 3 Results and Discussion

**Figure 1:**
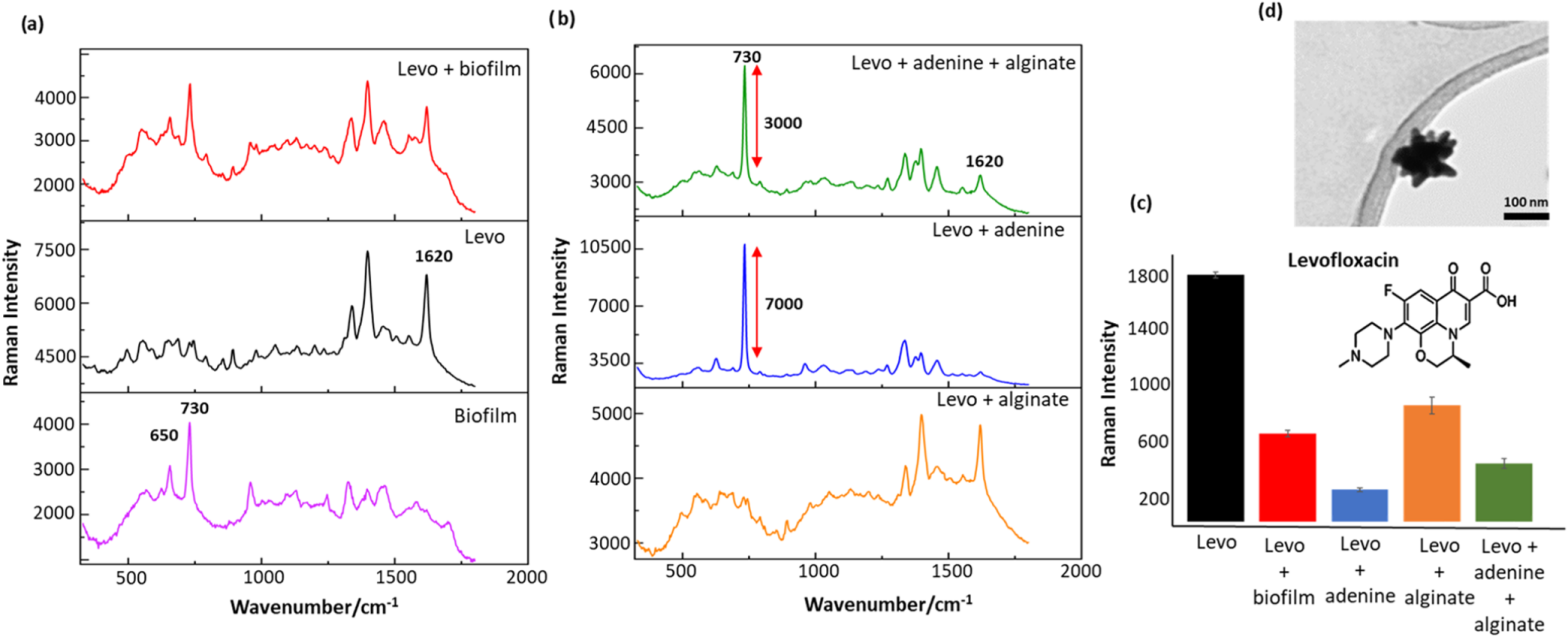
(a) Comparison of the spectra of biofilm only, Levo only and Levo within biofilm. (b) Comparison of the spectra of Levo in the presence of alginate, adenine and a mixture of adenine and alginate. All spectra are the average of four technical replicates recorded on the same day using the same colloid batch. (c) Comparison of the average Raman intensity of Levo only and Levo in the presence of biofilm, alginate, adenine and a mixture of adenine and alginate. (d) TEM image of the NS colloid. Scale bar = 100 nm.

In this work the studies were carried out using *ex-situ* biofilm samples since this gives a model system which retains the complex multicomponent composition of the biofilm but removes effects associated with the physical heterogeneity and low viscosity of intact biofilm samples. To reduce the possibility that changes in aggregation state of the enhancing particles caused by interaction with the biofilm constituents could perturb the SERS enhancement factor (EF), and therefore the overall signal intensity, gold nanostars were used as the substrates since they have previously been shown to provide the required enhancement without the need to form particle aggregates.^12^ The NS comprise a central metal core from which multiple sharp spikes protrude (Figure 1d). The spikes are the key element of these substrates, which serve as efficient nanoantennae. The electromagnetic field is expected to be very high at the end of each tip, giving rise to multiple intrinsic SERS hot spots within a single nanoparticle.^17^ In agreement with a previous report,^12^ these NS had an average size of 150 nm. They have a broad UV–Vis extinction band which centered at 680 nm but has strong extinction at the 785 nm excitation wavelength used for the SERS experiments (Figure S1), ensuring strong plasmonic enhancement.

These NS are known to spontaneously aggregate, which is undesirable because it prevents them being dispersed within the samples and it also reduces the plasmonic enhancement they provide. This makes them challenging to store and handle but here the NS were stabilized by adding a low concentration of hydroxyethyl cellulose (HEC). Importantly, this polymer does not significantly alter the colloid’s viscosity or chemically adsorb onto the surface of the NS, thus preventing aggregation while leaving the surface of the particles free for binding.^12^

The target molecules chosen for this study were two chemically distinct broad-spectrum antibiotics. Levofloxacin (Levo) which is a fluoroquinolone and ampicillin (Amp), which is an aminopenicillin.^19,20^ Initial studies were carried out using Levo, which shows intense SERS bands at 1620 and 1395 cm^–1^. These have been assigned to a C=C vibration of the quinolone ring system and a combination of the quinoline ring with (COO−) symmetric stretching, respectively. These characteristic signals are clearly observed in the SERS signals of Levo in the absence of biofilm, as shown in Figure 1a. However, for all the measurements in this study, the peak at 1620 cm^–1^ was chosen to monitor the SERS signals of Levo since the 1395 cm^–1^ band overlaps with features in the biofilm spectrum (Figure 1a).

The antibiotic concentration used in this study was set to 10^–3^ mol dm^–3^ and was chosen for its clinical relevance. For comparison, biofilm studies with *S. aureus* (strain ATCC 6538, starting inocula of 10^9^ cfu/ml) report a minimum bactericidal concentration (MBC) of 9.6 mg/mL (2.7 × 10^–2^ mol dm^–3^), which is over 3 orders of magnitude higher than in simple culture conditions. This highlights the complexity of detecting and treating antibiotics within biofilm environments.^21^ However, the concentration is still within the range which can be detected by SERS in *es*-biofilm.

The spectrum of the biofilm alone shows numerous distinct peaks, the most obvious being those at 650 and 728 cm^–1^ which are associated with nucleic acids.^7^ Addition of Levo at 10^–3^ mol dm^–3^ gave spectra where the strong characteristic bands at 1395 and 1620 cm^–1^ grew to be stronger than those of biofilm matrix, although they were reduced in intensity by approximately 70% compared to equivalent bands in aqueous solution at the same concentration (Figure 1a,c).

While the experiments with the es-biofilm are important in establishing that the matrix does not prevent Levo detection in the appropriate analytical range, they do not allow for any detailed understanding of the factors which lead to the significant reduction in intensity in the presence of the biofilm. Attributing the lower signal intensity of Levo in the biofilm matrix to a specific cause is challenging considering the numerous different factors at play in this type of measurements. One possible explanation is the competition between Levo and the matrix components for binding to the metallic surface, which is a common problem when using SERS for direct detection of target analytes in complex biological environments. In particular, biological environments are rich in adenine molecules, proteins and polysaccharides that can adsorb to the surface of unmodified nanoparticles like the NS colloid used here.^22^ These components can either compete with the antibiotic molecules or hinder their access to the enhancing surface. Here, we have addressed this problem by carrying out experiments to measure the effect of the presence of appropriate concentrations of model biofilm constituents on the SERS detection of the target drug at the same fixed concentration of 10^–3^ mol dm^–3^.

The first biofilm component investigated as a potential interfering species was adenine, partly because it is known that e- DNA plays an important role in the structure of biofilms and also because the SERS spectra of our biofilm samples show a peak at 730 cm^–1^ which is characteristic of adenine.^7,16^ Although it is not known whether this adenine is present as a free nucleic acid, nucleotide or part of an oligonucleotide (e.g., e-DNA), the fact that the signal is observed suggests that Levo will need to compete with adenine of some form when it is being detected in the presence of biofilm. Experiments to measure the effect of adding adenine on the SERS signal of Levo were carried out with an initial adenine concentration of 10^–4^ mol dm^–3^ since this is the concentration which was previously determined to be present in *S. aureus* es-biofilm.^12^ The adenine addition resulted in a large (approximately 90%) reduction in the intensity of the Levo SERS signal (Figure 1b,c). This decrease can be attributed to the strong affinity of adenine to the metallic surface blocking the binding sites for Levo. However, the 90% reduction in Levo signals in the presence of adenine alone was significantly greater than the 70% reduction observed in the presence of *es*-biofilm which contained the same concentration of adenine (Figure 1a,c). This is surprising since it might be expected that the adenine would interfere with the Levo adsorption in the biofilm at least to the same extent that it did so in solution. Moreover, proteins and polysaccharides in the biofilm matrix would also be expected to block additional sites, causing even more reduction in signal intensity in the biofilm compared to the adenine-only sample. The reason for this unexpected behavior only became clear following the experiments on the other model biofilm components discussed below.

The next species which could also potentially interfere with the detection of the drug are the polysaccharides which are a major component of the biofilm. Here we employed alginate as a representative polysaccharide since it is known to be present in biofilms and is chemically similar to the other polysaccharides which are also present.^23^ In this case it was found that the SERS signal of Levo was reduced by ca. 50% (Figure 1b,c) in the presence of alginate. By analogy with the adenine effect discussed above, the most obvious explanation for the reduction in the Levo signal in the presence of alginate is that the alginate binds to the surface, blocking access of Levo to the enhancing surface. While there are no extensively reported studies on the binding of alginate to the surface of the metal colloid, here we investigated the possibility using experiments where the NS were coated with citrate ions to give a displaceable surface layer with easily identified marker bands. It was found that when alginate was added to a mixture of citrate-NS colloid, alginate was able to remove the citrate and bind to the surface of the nanoparticles, as indicated by the appearance of alginate SERS signals including peaks at 930, 1128, and 1300 cm^–1^ in the citrate-treated sample after addition of alginate.^24^ (see Figure S2 of Supporting Information for spectra).

Given the clear evidence that both adenine and alginate can bind to the NS and that each individually causes a significant reduction in the Levo SERS signal (by 90 and 50%, respectively) it would be expected that the SERS signal of Levo would be reduced even further in media where both adenine and alginate are present. However, as shown in Figure 1b,c introduction of alginate along with adenine actually increases the Levo signal over that obtained with adenine alone. The effect is significant, with the signal increasing from 218 ± 14 to 437 ± 38 cts. There are numerous potential explanations for this observation, but it is easy to rule out the possibility that it is associated with changes in aggregation because no spectral shifts were observed in the NS UV–vis spectra when they were recorded for the NS in the presence of the biofilm components (adenine, alginate, and adenine-alginate, see Figure S1). In order to eliminate the possibility that it was associated with the properties of Levo, the measurements were repeated with ampicillin (Amp) which has a very different chemical structure.

**Figure 2:**
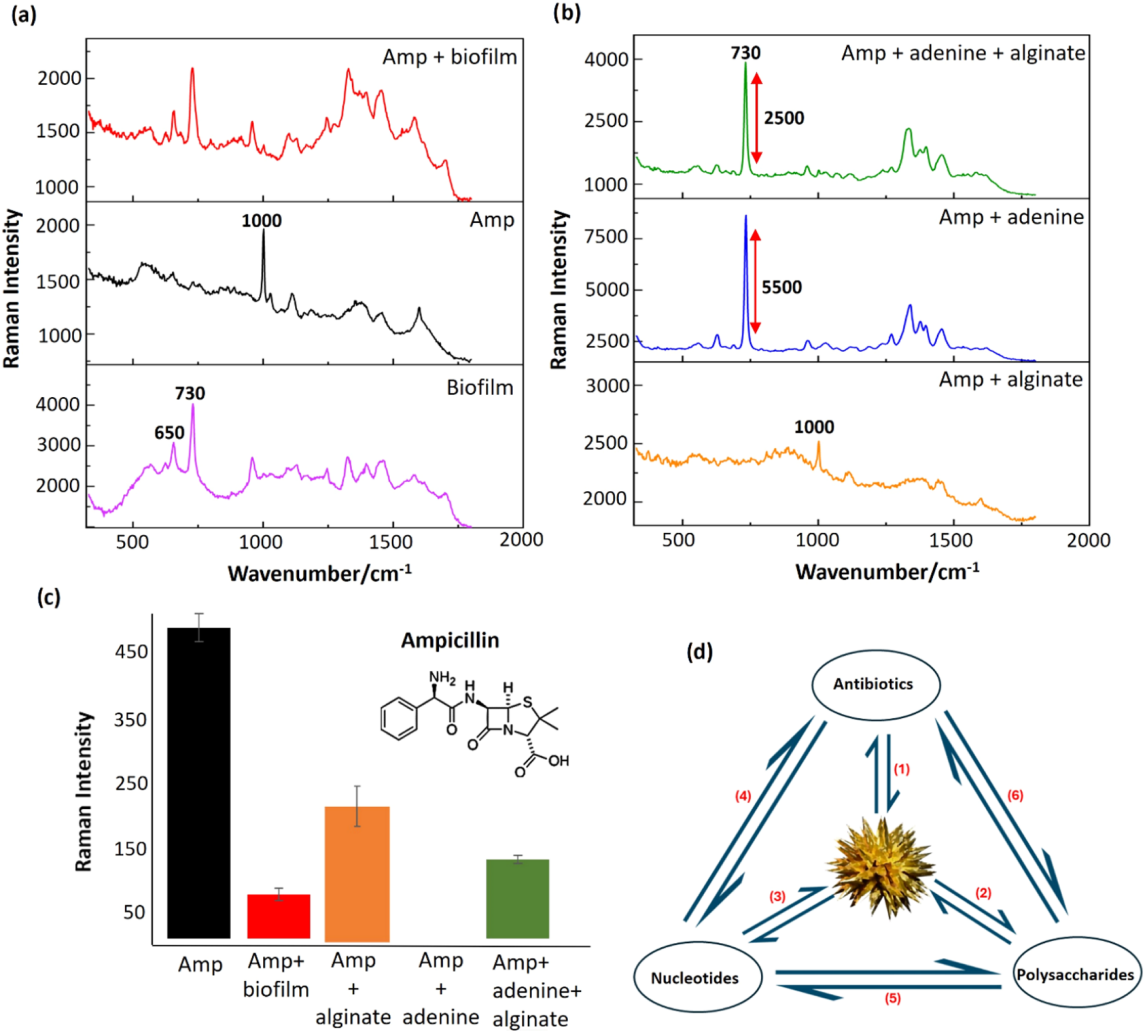
(a) Comparison of the SERS spectra of Amp only, biofilm only and Amp within biofilm. (b) Comparison of the spectra of Amp in the presence of alginate, adenine and a mixture of adenine and alginate. All spectra are the average of four technical replicates recorded on the same day using the same colloid batch. (c) Comparison of the average Raman intensity of Amp only and Amp in the presence of biofilm, alginate, adenine and a mixture of adenine and alginate. (d) Illustration of the possible interactions in the NS-Drug-Nucleotide-Polysaccharide System.

Amp gives weaker SERS signals than Levo but it does have a characteristic strong phenyl ring vibration at 1000 cm^–1^ which is an uncongested spectral region.^20^Figure 2a,c show that the aqueous solution Amp SERS signal was reduced by 90% in the presence of *es*-biofilm, which is even higher than the 70% reduction recorded for Levo. The individual effects of alginate and adenine were again both observed to reduce the Amp signal. With alginate, the Amp signal dropped by 50%, similar to that observed for Levo. With the adenine-only sample, the Amp signal fell to an undetectably low value, matching the large decrease (90%) found for Levo (Figure 2b,c). Importantly, the effect of alginate was also to reverse the impact of adenine on the signals of the antibiotic, so that it was possible to obtain Amp signals in samples with 10^–5^ mol dm^–3^ adenine, provided alginate was also present (Figure 2b,c).

Since the unexpected effect of the combination of adenine and alginate on the SERS signals was found for both Levo and Amp, the origin of the effect is not molecule specific but is characteristic of the system as a whole. This is a complex problem, since in a system composed of NS, drug molecules, adenine (nucleotides) and alginate (polysaccharides) there are six different possible pairwise interactions, as illustrated in Figure 2d. Equilibria (1–3) can be measured by SERS on pure solutions. The binary experiments discussed above probe three equilibria simultaneously, for example experiments with Levo plus alginate involve equilibria (1), (2) and (6), so the loss of the intensity of the Levo band can be interpreted as perturbation of (1) by the polysaccharide, which could be either through blocking the surface (equilibrium (2)) or binding the drug and preventing it reaching the surface (equilibrium (6)). Since the effect is independent of the drug, the latter explanation is less likely (although still possible if both drugs act similarly), so we can assign it to blocking of the surface, as was discussed above. Similar arguments apply for the binary drug/adenine experiments, where equilibrium (3) dominates. This means that we can reasonably discount equilibria (4) and (1).

**Figure 3:**
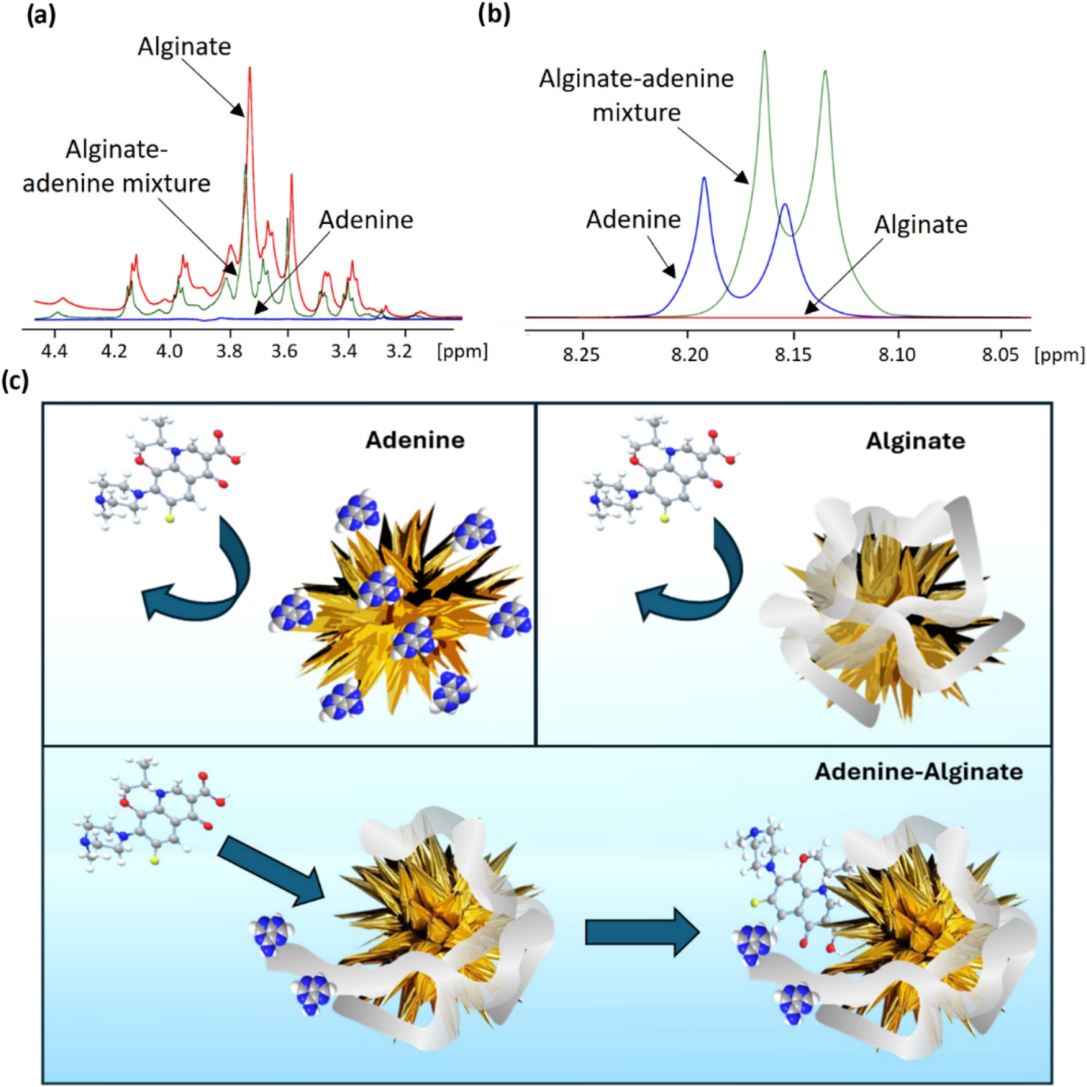
Comparison of ^1^H NMR spectra of alginate (red) adenine (blue) and adenine-alginate mixture (green) (a) Indicates shift in the spectra of the adenine-alginate sample compared to alginate only sample. (b) Indicates shift in the adenine- alginate sample compared to adenine only sample. (c) Illustration of Levo binding to NS in the presence of adenine, alginate and a mixture of adenine and alginate.

The final binary experiment adenine/alginate simply shows loss of adenine bands on adding alginate (see Figure S3 of Supporting Information for spectra) but it is not possible to determine if this is due to blocking of surface sites (2) or adenine interacting directly with alginate (5). To address this question, ^1^H NMR measurements were conducted to determine if binding between adenine and alginate does indeed occur in solution. The resulting ^1^H NMR spectra of adenine, alginate, and their mixture were compared to detect any changes indicative of interaction between adenine and alginate relative to the spectra of the pure samples. While the individual spectra of adenine and alginate were found to be consistent with the literature, significant changes occurred in both adenine and alginate spectra upon mixing.^25,26^ Notably, clear shifts were observed in the adenine proton peaks found at δH ∼8.0–8.2 ppm upon the addition of alginate (Figure 3b). Similarly, in the ^1^H NMR spectra of sodium alginate, which typically consists of peaks in the region δH ∼3.2–4.4 ppm corresponding to the proton resonances of mannuronate and guluronate (monomers comprising alginate),^25^ significant shifts in the signals were observed upon adenine addition (Figure 3a).

Changes in ^1^H NMR signals, including shifts in the chemical shifts of ^1^H NMR, are a well-known indicator of interaction between molecules and complex formation. For example, the investigation of lectin interaction with sugar (chitotriose) using 1D ^1^H NMR spectroscopy demonstrated significant shifts in the chemical shifts of all the lectin signals in the presence of the sugar, indicative of sugar-protein complex formation.^27^ Consequently, the observed shift in ^1^H NMR signals of alginate and adenine in the mixture would suggest a strong interaction between them, possibly through the formation of hydrogen bonds between the −OH groups of alginate’s carboxylic acid groups and the nitrogen atoms in adenine. Binding of carboxylic acid groups from benzoic acid, adipic acid and salicylic acid and adenine has been documented in the literature.^28^ Indeed, it has been found that binding of monosaccharides and disaccharides to adenine prevents the precipitation of crystalline adenine from aqueous solutions.^29^ Similarly, in this work it was found that 0.5% (w/v) adenine solutions in the presence of 1% (w/v) alginate could be prepared and used without any noticeable precipitation, whereas aqueous adenine samples needed to be analyzed at 0.1% to avoid precipitation and distortion of their NMR spectra.

This data now allows us to account for the ternary drug/adenine/alginate data which showed the unexpectedly large drug signals and involves all of the equilibria discussed above in a single experiment. In this case the addition of alginate to a system with just drug and adenine would be expected to reduce the signals of both since the alginate will adsorb to the surface ((equilibrium 2) and thus hinder access for both the drug and adenine (equilibria 1 and 3)). However, in addition to this blocking effect, the alginate will also reduce the amount of free adenine available to bind to the surface of the nanoparticles due to the strong adenine/alginate interactions (5) discussed above. This would be expected to further reduce adenine binding to the nanoparticle surface and as a result allow more drug to adsorb in its place (Figure 3c). The net result is that this model would predict that alginate addition would reduce the adenine signal while simultaneously increasing that of the Levo. This is confirmed in the experiment where close examination of the spectra in Figures 1b and 2b shows that adding alginate alongside adenine not only unexpectedly increases the drug bands but also reduces the adenine signal at 730 cm^–1^ by approximately 50% in both Levo and Amp samples.

**Figure 4:**
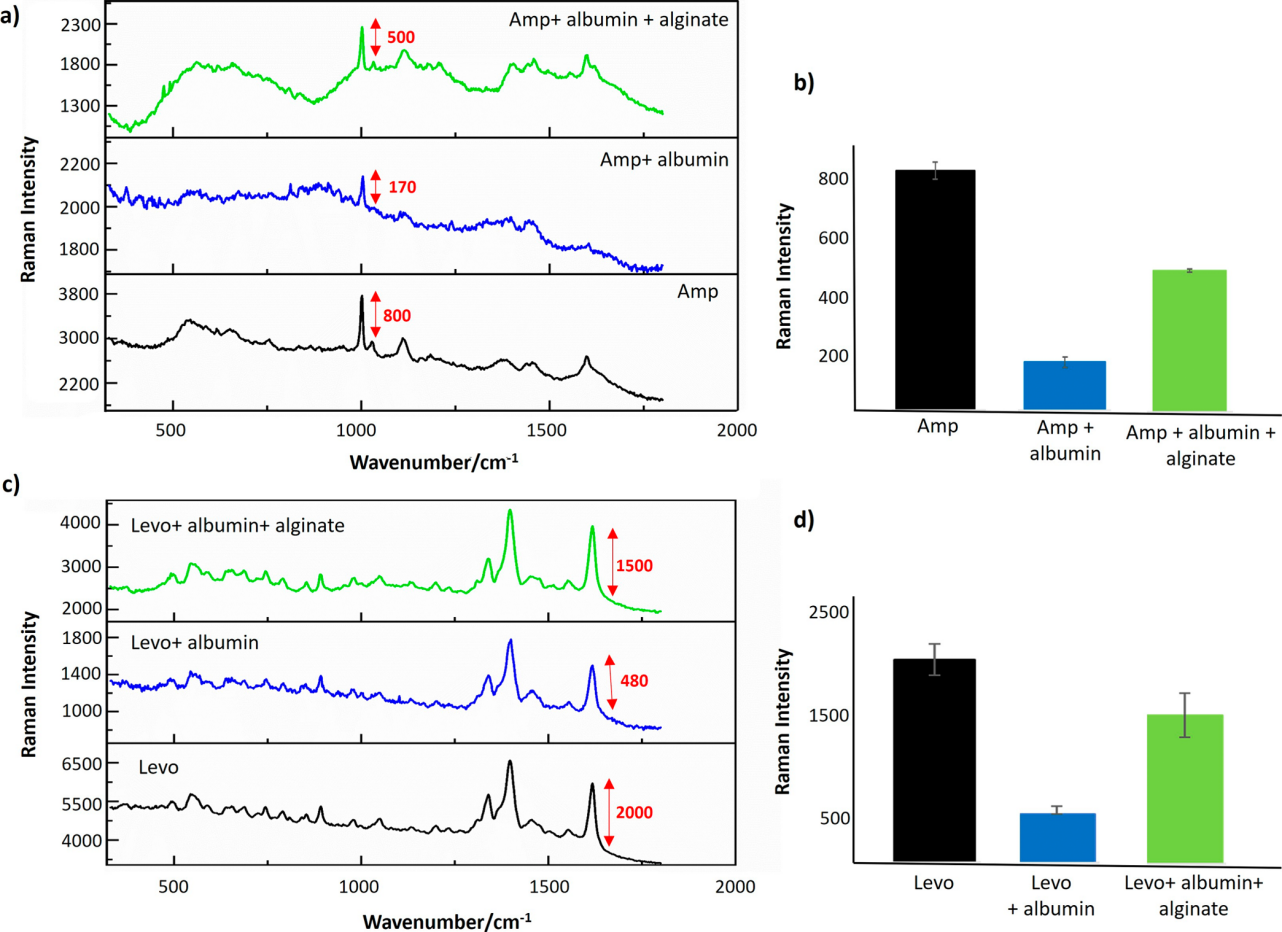
(a) Comparison of the spectra of Amp only, Amp in the presence of albumin, and in a mixture of albumin and alginate. (b) Corresponding Raman intensity comparison of Amp. (c) Comparison of the spectra of Levo only, Levo in the presence of albumin, and in a mixture of albumin and alginate (d) Corresponding Raman intensity comparison of Levo. All spectra are the average of four technical replicates recorded on the same day using the same colloid batch.

Since the data above probe the effect which adenine, an additional component within the biofilm, affects the polysaccharide/drug/surface interactions, it is useful to extend this work to investigate the effect of protein, another additional biofilm component, on the same set of interactions. In these experiments bovine serum albumin was used as the model protein and albumin or a mixture of albumin and alginate were added to the NS and allowed to interact with the surface before the addition of antibiotics. As shown in Figure 4c,d the SERS signal of Levo at 1620 cm^–1^ was reduced to ca. 25% of its original value in the presence of albumin, which is consistent with protein adsorption hindering access of the drug to the enhancing surface, as has been widely reported for other systems.^10^ However, as was the case for adenine, addition of alginate reduced the blocking effect of the protein and in the mixture the drug signal was still 75% of the original value. Similar results were observed for Amp, with the SERS signal at 1000 cm^–1^ being approximately 70% higher in the presence of albumin and alginate compared to the albumin only sample (Figure 4a,b).

The albumin data can be interpreted in a similar way to that for the adenine experiments i.e., by postulating that the unexpectedly large drug signal found when albumin and alginate are present is due to a reduction in the availability of free albumin and/or alginate molecules to block the surface of the NS. In this case the possibility of binding between albumin and alginate allowing increased access to the surface is supported by the literature where the electrostatic attraction between the positively charged segments of the protein and the carboxyl groups of the alginate has already been reported.^30^

The observations above lend support to the general observation that in complex mixed component systems such as biofilms, interpretation of data from nanoparticle probes need to consider the sample as a complex system where numerous intermolecular interactions are coupled together, which can lead to unexpected consequences. In a general sense the observation of interactions between the components in a biofilm is not surprising since they are known to play an important role in biofilms, for example interactions between the polysaccharides and e-DNA have been found to be essential in maintaining the structure of the film.^16^ Similarly, interactions between individual constituents and the surface of the nanoprobes have also been explored in considerable detail for example, in the detection of small molecule drugs, or investigating the formation of protein coronas around nanoparticles.^14,19^ The current study extends the existing studies by showing that in these complex systems the signals depend not only on the direct interactions of targets with the surface but also on the secondary effects of intermolecular interactions, which are present within the system but not directly involved in surface binding. In this case, interactions between the polysaccharides and nucleosides or protein components are apparently entirely separate from those between the drug and nanoparticle surface, but they still have the ability to change the result of the measurement because all these equilibria are coupled within these multicomponent systems. Although this is a general observation, it does have particular relevance in the case of biofilms, since the fact that the interactions between potentially interfering components partly cancel out their detrimental effects has the fortunate result that antibiotic drugs can be detected at clinically relevant concentrations.

## 4 Conclusions

The structure of even *es*-biofilm is complex and difficult to study due to the presence of high molecular weight biopolymers. SERS provides an excellent molecularly specific molecular probe that can interrogate the local environment around the enhancing particles *in situ* and can also be used in studies where the complex components are modeled by simpler components such as alginate (polysaccharides), albumin (proteins) and adenine (nucleic acids and polynucleic acids). Since some of the components in the biofilm such as the adenine and alginate give their own characteristic SERS spectra, they can be monitored directly while indirect experiments can be used to infer the effect of interactions between the constituents in the biofilm by monitoring probe molecules on the enhancing particles.

In the current study it was clear that in order to interpret the results of measurements of any of the components within the biofilm (including added small molecules drug compounds) it is essential to consider how the presence of other compounds and the interactions between them can perturb the results. This is not simply because other constituents may compete for surface sites or block access to the surface (although these are important effects) but also because interactions in the bulk which are completely independent of any surface have a secondary effect of changing the way in which the surface can interact with its local environment. In the current study, the data for the detection of both Levo and Amp in simple solutions of the model components showed that these compounds decreased the SERS signals of the drugs to a significant degree, but combinations of these components led to drug signals that increased rather than decreased in intensity. This observation is important for quantitative studies of biofilms since it suggests that the effect of the matrix may be significantly less than would have been expected on the basis of model studies. It will also help to suppress the fluctuations in signals associated with changes relative proportions of the various interfering species present in each biofilm sample since each will tend to reduce the individual effects of the others. Reduction of these matrix effects will be essential for quantitative measurements of drug compounds within intact biofilms since in such experiments there is no possibility of correcting for them using standard addition in the way that is can be used for *es*-biofilm studies.

Finally, it is useful to note that while the model systems studied here can capture important features of the biofilm matrix, intact biofilms are even more complex due to the presence of active microbial communities, which introduce additional interactions beyond those observed in these models. Metabolic activity, enzymatic processes and dynamic EPS production can further influence nanoparticle interactions and drug-binding dynamics, potentially altering the observed equilibria. The next challenge is to extend the current studies to investigate the effect of these dynamic processes on the properties of intact biofilms.

## Supporting Information Available

UV–vis extinction spectrum; TEM images; particle size distribution and SERS spectra of various components (PDF)
