# Supplementary material for: Probing the Coupled Equilibria between Metal Nanoparticles, Antibiotics and Components of the Extracellular Matrix in Biofilms with SERS
Source: Biomacromolecules. 2025 Apr 7;26(5):2900–8. doi: 10.1021/acs.biomac.4c01707 (PMC12076487; doi:10.1021/acs.biomac.4c01707)
Supplement: Supplementary file 1 — bm4c01707_si_001.pdf [file bm4c01707_si_001.pdf]

Supplementary Information

Probing the Coupled Equilibria Between Metal Nanoparticles, Antibiotics and Components of the Extracellular Matrix in Biofilms with SERS.

Wafaa Aljuhani[a], Matthew P. Wylie[b], Rudra N. Purusottam[a], Colin P. McCoy[b], Steven E. J. Bell[a]*

a. School of Chemistry and Chemical Engineering, Queen's University Belfast, BT9 5AG Belfast, United Kingdom.

b. School of Pharmacy, Queen's University Belfast, BT9 7BL Belfast, United Kingdom.

Corresponding author:

Steven E.J. Bell, email: s.bell@qub.ac.uk

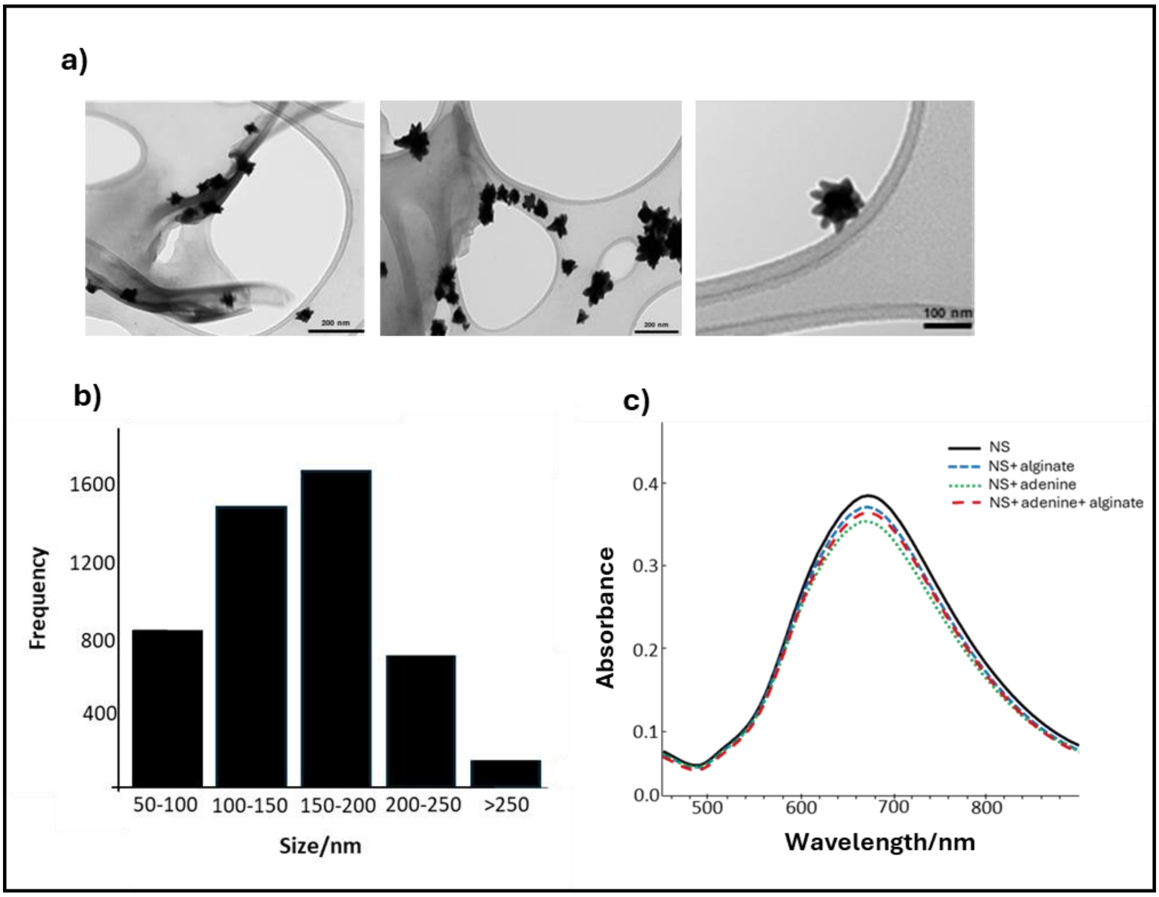

Figure S1. (a) TEM images of nanostars (NS). (b) Particle size distribution of NS obtained using a Nanosight. (c) UV-vis extinction spectra of NS alone and in the presence of alginate, adenine and adenine-alginate.

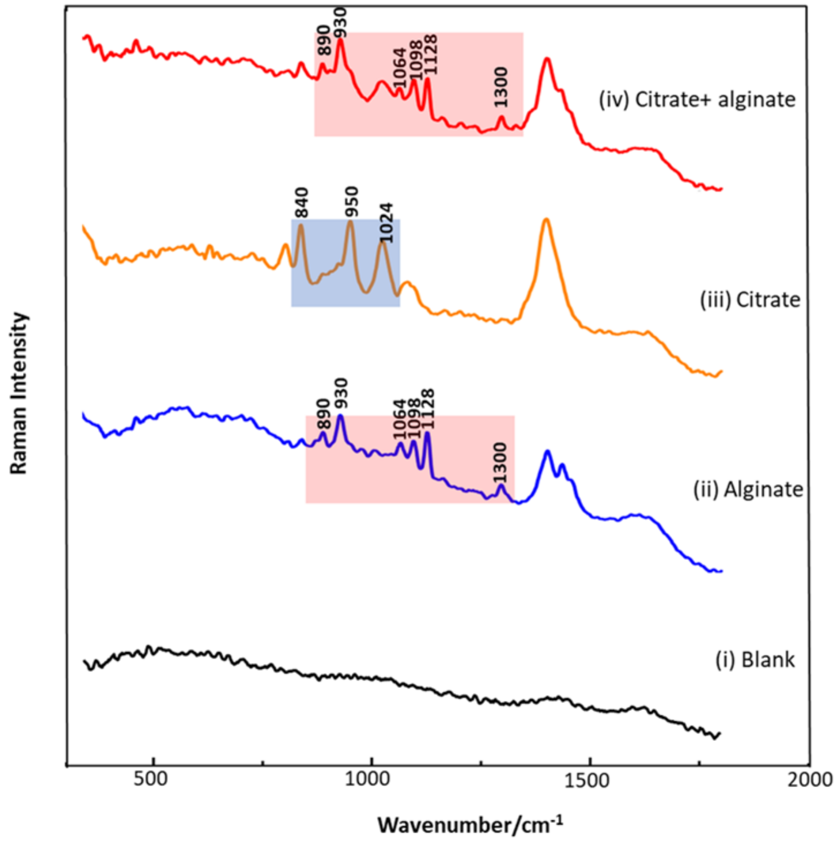

Figure S2. SERS spectra of (i) blank NS colloid, (ii) alginate only, (iii) citrate only and (iv) alginate-citrate mixture.

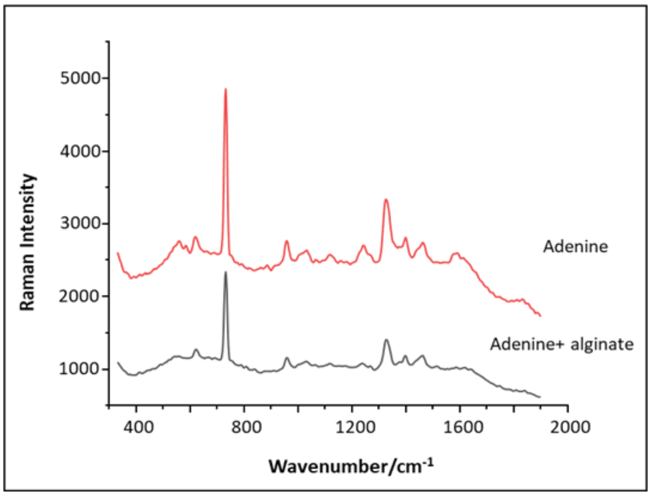

Figure S3. SERS spectra of adenine only and adenine in presence of alginate.
